# Crystal structure of (*E*)-*N*′-(4-chloro­benzyl­idene)-4-methyl­benzene­sulfono­hydrazide: a hexa­gonal polymorph

**DOI:** 10.1107/S1600536814023721

**Published:** 2014-11-12

**Authors:** J. Balaji, J. John Francis Xavier, S. Prabu, P. Srinivasan

**Affiliations:** a Department of Physics, University College of Engineering Panruti, Tamil Nadu 607 106, India; b Department of Chemistry, University College of Engineering Panruti, Tamil Nadu 607 106, India

**Keywords:** crystal structure, hydrazones, sulfono­hydrazide, Schiff base, helical chains, hydrogen bonding.

## Abstract

The title compound, C_14_H_13_ClN_2_O_2_S, crystallized in the enanti­omorphic defining hexa­gonal space group *P*6_1_ [Flack parameter = −0.02 (7)]. The partially hydrated form of the same compound, crystallizing in the triclinic space group *P*-1, has been reported previously [Kia *et al.* (2009*b*). *Acta Cryst.* E**65**, o1119], as has the crystal structure of the bromo derivative, also crystallizing in the space group *P*-1 [Kia *et al.* (2009*a*). *Acta Cryst.* E**65**, o821]. The title mol­ecule is non-planar with the planes of the benzene rings being inclined to one another by 76.62 (13)°, and has an *E* conformation about the C=N bond. In the crystal, mol­ecules are linked *via* N—H⋯O hydrogen bonds forming 6_1_ helical chains running along [001]. The chains are linked *via* C—H⋯O hydrogen bonds, C—H⋯π inter­actions and short Cl⋯O [3.015 (3) Å] inter­actions, forming a three-dimensional structure.

## Related literature   

For the biological activities of hydrazones, see: Ajani *et al.* (2010 ▶). For the crystal structure of the triclinic polymorph, which crystallized with two independent mol­ecules in the asymmetric unit, one of which was disordered, and with 0.15 of a water mol­ecule, see: Kia *et al.* (2009*b* ▶). For the crystal structure of the bromo derivative, also crystallizing in space group *P* 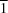, see: Kia *et al.* (2009*a* ▶).
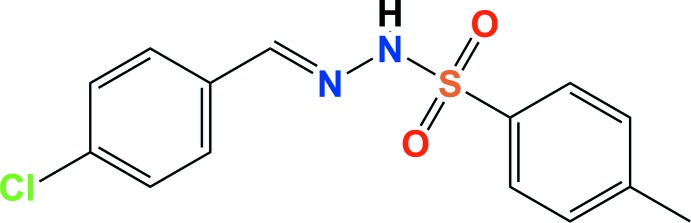


## Experimental   

### Crystal data  

C_14_H_13_ClN_2_O_2_S
*M* *_r_* = 308.77
Hexagonal, 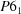

*a* = 10.8907 (3) Å
*c* = 21.4542 (7) Å
*V* = 2203.71 (11) Å^3^
*Z* = 6
Mo *K*α radiation
μ = 0.40 mm^−1^
*T* = 293 K
0.35 × 0.30 × 0.25 mm

### Data collection  

Bruker Kappa APEXII CCD diffractometer
Absorption correction: multi-scan (*SADABS*; Sheldrick, 1996 ▶) *T*_min_ = 0.871, *T*_max_ = 0.910
22095 measured reflections
2586 independent reflections
2345 reflections with *I* > 2σ(*I*)
*R*_int_ = 0.027

### Refinement  

*R*[*F* ^2^ > 2σ(*F* ^2^)] = 0.029
*wR*(*F* ^2^) = 0.072
*S* = 1.04
2586 reflections
186 parameters
2 restraints
H atoms treated by a mixture of independent and constrained refinement
Δρ_max_ = 0.12 e Å^−3^
Δρ_min_ = −0.16 e Å^−3^
Absolute structure: Flack (1983 ▶), 1257 Friedel pairs
Absolute structure parameter: −0.02 (7)

### 

Data collection: *APEX2* (Bruker, 2004 ▶); cell refinement: *APEX2* and *SAINT* (Bruker, 2004 ▶); data reduction: *SAINT* and *XPREP* (Bruker, 2004 ▶); program(s) used to solve structure: *SIR92* (Altomare *et al.*, 1993 ▶); program(s) used to refine structure: *SHELXL97* (Sheldrick, 2008 ▶); molecular graphics: *ORTEP-3 for Windows* (Farrugia, 2012 ▶) and *Mercury* (Macrae *et al.*, 2008 ▶); software used to prepare material for publication: *SHELXL97* and *PLATON* (Spek, 2009 ▶).

## Supplementary Material

Crystal structure: contains datablock(s) I, New_Global_Publ_Block. DOI: 10.1107/S1600536814023721/su2800sup1.cif

Structure factors: contains datablock(s) I. DOI: 10.1107/S1600536814023721/su2800Isup2.hkl

Supporting information file. DOI: 10.1107/S1600536814023721/su2800Isup3.cml

**Fig. 1.**
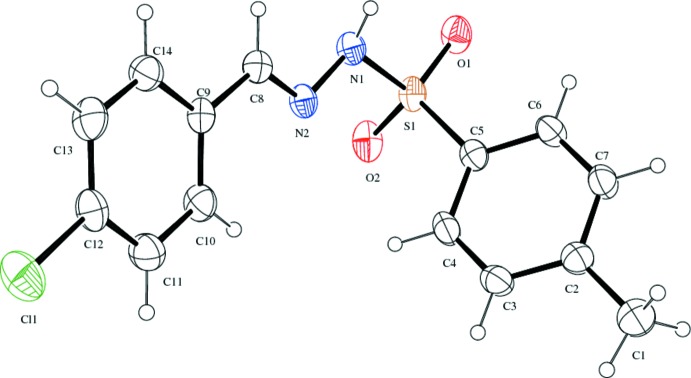
The molecular structure of the title compound, with atom labelling. The displacement ellipsoids are drawn at the 30% probability level.

**Fig. 2.**
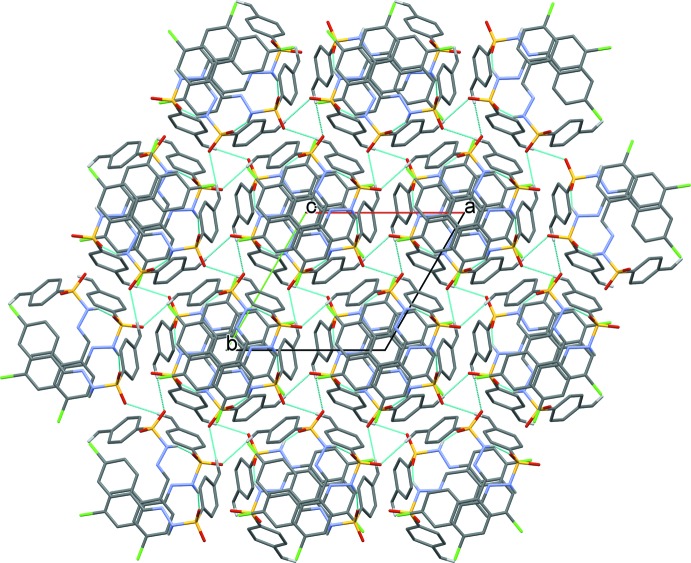
A view along the c axis of the crystal packing of the title compound. The N—H···O and C—H···O hydrogen bonds are indicated by dashed lines (see Table 1 for details; H atoms not involved in these interactions have been omitted for clarity).

. DOI: 10.1107/S1600536814023721/su2800fig1.tif
The mol­ecular structure of the title compound, with atom labelling. The displacement ellipsoids are drawn at the 30% probability level.

c . DOI: 10.1107/S1600536814023721/su2800fig2.tif
A view along the *c* axis of the crystal packing of the title compound. The N—H⋯O and C—H⋯O hydrogen bonds are indicated by dashed lines (see Table 1 for details; H atoms not involved in these inter­actions have been omitted for clarity).

CCDC reference: 1031289

Additional supporting information:  crystallographic information; 3D view; checkCIF report

## Figures and Tables

**Table 1:** Hydrogen-bond geometry (, ) *Cg* is the centroid of the C2C7 ring.

*D*H*A*	*D*H	H*A*	*D* *A*	*D*H*A*
N1H1O2^i^	0.88(2)	2.13(2)	2.952(3)	156(2)
C1H1*A*O1^ii^	0.96	2.55	3.496(5)	169
C13H13*Cg* ^iii^	0.93	2.94	3.823(3)	160

Symmetry codes: (i) 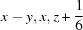; (ii) 
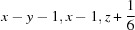
; (iii) 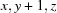.

## supplementary crystallographic information

### S1. Comment

The title compound was obtained by a Schiff base condensation reaction between 4-chlorobenzaldehyde and tosyl hydrazide. Hydrazones have received much attention recently due to their biological activities (Ajani *et al.*, 2010). The crystal structure of the triclinic polymorph, that crystallized with two independent molecules in the asymmetric unit, one of which was disordered, and with 0.15 of a water molecule, has been reported (Kia *et al.*, 2009b), as has the crystal structure of the bromo derivative, also crystallizing in space group P1 (Kia *et al.*, 2009a).

The hydrazone molecule, Fig. 1, exists in a *trans* or *E* confirmation with respect to the C8═N2 bond. The dihedral angle between the (C2—C7) and (C9—C14) benzene rings is 76.62 (13) °. In the triclininc polymorph (Kia *et al.*, 2009b) the same angle is 84.96 (11) ° (and 71.1 (3) ° for the disordered molecule), and 82.39 (13) ° for the bromo derivative (Kia *et al.*, 2009a).

In the crystal, molecules are linked *via* N—H···O hydrogen bonds forming 6_1_ helical chains running along the *c* axis direction (Table 1 and Fig 2). The chains are linked *via* C-H···O hydrogen bonds, and a short Cl···O2^i^ [3.015 (3) Å; symmetry code: (i) x-y+1, x, z+1/6] interaction and a C-H···π interaction, forming a three-dimensional structure (Table 1 and Fig. 2).

### S2. Experimental

4-chlorobenzaldehyde (0.140 g, 1 mmol) and tosyl hydrazide (0.186 g, 1 mmol) were dissolved in ethanol and chloroform (4:1). The reaction mixture was heated under reflux for 3 h and cooled gradually to room temperature. Prismatic colourless crystals were obtained by slow evaporation of an ethanol solution at room temperature.

### S3. Refinement

The NH H atom was located in a difference Fourier map and freely refined. The C-bound H atoms were positioned geometrically and treated as riding on their parent atoms with C—H = 0.93 Å (aromatic) and 0.96 Å (methyl) and with *U*_iso_(H) = 1.5*U*_eq_(C) for the methyl H atoms and = 1.2*U*_eq_(C) for other H atoms.

### Crystal data

**Table:**

C_14_H_13_ClN_2_O_2_S	*D*_x_ = 1.396 Mg m^−^^3^
*M**_r_* = 308.77	Mo *K*α radiation, λ = 0.71073 Å
Hexagonal, *P*6_1_	Cell parameters from 9375 reflections
Hall symbol: P 61	θ = 2.4–25.8°
*a* = 10.8907 (3) Å	µ = 0.40 mm^−^^1^
*c* = 21.4542 (7) Å	*T* = 293 K
*V* = 2203.71 (11) Å^3^	Block, yellow
*Z* = 6	0.35 × 0.30 × 0.25 mm
*F*(000) = 960	

### Data collection

**Table:**

Bruker Kappa APEXII CCD diffractometer	2586 independent reflections
Radiation source: fine-focus sealed tube	2345 reflections with *I* > 2σ(*I*)
Graphite monochromator	*R*_int_ = 0.027
ω and φ scan	θ_max_ = 25.0°, θ_min_ = 2.2°
Absorption correction: multi-scan (*SADABS*; Sheldrick, 1996)	*h* = −12→12
*T*_min_ = 0.871, *T*_max_ = 0.910	*k* = −12→12
22095 measured reflections	*l* = −25→25

### Refinement

**Table:**

Refinement on *F*^2^	Hydrogen site location: inferred from neighbouring sites
Least-squares matrix: full	H atoms treated by a mixture of independent and constrained refinement
*R*[*F*^2^ > 2σ(*F*^2^)] = 0.029	*w* = 1/[σ^2^(*F*_o_^2^) + (0.0306*P*)^2^ + 0.7106*P*] where *P* = (*F*_o_^2^ + 2*F*_c_^2^)/3
*wR*(*F*^2^) = 0.072	(Δ/σ)_max_ = 0.001
*S* = 1.04	Δρ_max_ = 0.12 e Å^−^^3^
2586 reflections	Δρ_min_ = −0.16 e Å^−^^3^
186 parameters	Extinction correction: *SHELXL97* (Sheldrick, 2008), Fc^*^=kFc[1+0.001xFc^2^λ^3^/sin(2θ)]^-1/4^
2 restraints	Extinction coefficient: 0.0026 (4)
Primary atom site location: structure-invariant direct methods	Absolute structure: Flack (1983), 1257 Friedel pairs
Secondary atom site location: difference Fourier map	Absolute structure parameter: −0.02 (7)

### Special details

**Table:**

Geometry. All e.s.d.'s (except the e.s.d. in the dihedral angle between two l.s. planes) are estimated using the full covariance matrix. The cell e.s.d.'s are taken into account individually in the estimation of e.s.d.'s in distances, angles and torsion angles; correlations between e.s.d.'s in cell parameters are only used when they are defined by crystal symmetry. An approximate (isotropic) treatment of cell e.s.d.'s is used for estimating e.s.d.'s involving l.s. planes.
Refinement. Refinement of *F*^2^ against ALL reflections. The weighted *R*-factor *wR* and goodness of fit *S* are based on *F*^2^, conventional *R*-factors *R* are based on *F*, with *F* set to zero for negative *F*^2^. The threshold expression of *F*^2^ > σ(*F*^2^) is used only for calculating *R*-factors(gt) *etc*. and is not relevant to the choice of reflections for refinement. *R*-factors based on *F*^2^ are statistically about twice as large as those based on *F*, and *R*- factors based on ALL data will be even larger.

### Fractional atomic coordinates and isotropic or equivalent isotropic displacement parameters (Å^2^)

**Table:**

	*x*	*y*	*z*	*U*_iso_*/*U*_eq_	
C1	−0.1026 (3)	−0.5080 (4)	0.94696 (15)	0.0727 (9)	
H1A	−0.2001	−0.5364	0.9392	0.109*	
H1B	−0.0699	−0.4482	0.9831	0.109*	
H1C	−0.0942	−0.5906	0.9539	0.109*	
C2	−0.0151 (3)	−0.4286 (3)	0.89196 (13)	0.0480 (6)	
C3	−0.0778 (3)	−0.4059 (3)	0.84061 (13)	0.0507 (6)	
H3	−0.1751	−0.4410	0.8405	0.061*	
C4	0.0019 (3)	−0.3321 (3)	0.78968 (12)	0.0496 (6)	
H4	−0.0415	−0.3180	0.7553	0.060*	
C5	0.1458 (2)	−0.2795 (2)	0.79003 (11)	0.0419 (5)	
C6	0.2103 (3)	−0.3013 (3)	0.84070 (12)	0.0483 (6)	
H6	0.3077	−0.2657	0.8408	0.058*	
C7	0.1296 (3)	−0.3758 (3)	0.89098 (13)	0.0514 (6)	
H7	0.1730	−0.3911	0.9250	0.062*	
C8	0.1633 (3)	0.1027 (3)	0.74279 (11)	0.0426 (5)	
H8	0.2543	0.1797	0.7474	0.051*	
C9	0.0430 (3)	0.1244 (3)	0.74794 (10)	0.0415 (5)	
C10	−0.0947 (3)	0.0146 (3)	0.74508 (13)	0.0520 (6)	
H10	−0.1127	−0.0766	0.7367	0.062*	
C11	−0.2070 (3)	0.0381 (3)	0.75456 (13)	0.0579 (7)	
H11	−0.3000	−0.0366	0.7526	0.069*	
C12	−0.1789 (3)	0.1731 (3)	0.76682 (12)	0.0522 (6)	
C13	−0.0439 (3)	0.2844 (3)	0.76863 (13)	0.0574 (7)	
H13	−0.0266	0.3758	0.7763	0.069*	
C14	0.0664 (3)	0.2595 (3)	0.75898 (12)	0.0516 (6)	
H14	0.1589	0.3353	0.7599	0.062*	
O1	0.3852 (2)	−0.1677 (2)	0.73010 (9)	0.0597 (5)	
O2	0.1652 (2)	−0.2455 (2)	0.67125 (8)	0.0605 (5)	
S1	0.24764 (7)	−0.18427 (7)	0.72562 (3)	0.04549 (16)	
Cl1	−0.31836 (9)	0.20068 (10)	0.78326 (4)	0.0779 (3)	
N1	0.2735 (2)	−0.0230 (2)	0.73014 (10)	0.0445 (5)	
N2	0.1474 (2)	−0.0187 (2)	0.73212 (9)	0.0424 (5)	
H1	0.337 (2)	0.033 (2)	0.7573 (10)	0.050 (8)*	

### Atomic displacement parameters (Å^2^)

**Table:**

	*U*^11^	*U*^22^	*U*^33^	*U*^12^	*U*^13^	*U*^23^
C1	0.0601 (19)	0.084 (2)	0.0681 (19)	0.0315 (17)	0.0065 (15)	0.0170 (17)
C2	0.0489 (14)	0.0454 (14)	0.0498 (13)	0.0237 (12)	−0.0031 (13)	−0.0033 (12)
C3	0.0402 (14)	0.0510 (15)	0.0621 (16)	0.0238 (12)	−0.0056 (12)	−0.0032 (13)
C4	0.0518 (15)	0.0533 (15)	0.0512 (14)	0.0319 (13)	−0.0149 (12)	−0.0028 (13)
C5	0.0472 (14)	0.0423 (13)	0.0434 (13)	0.0278 (12)	−0.0050 (11)	−0.0061 (11)
C6	0.0403 (14)	0.0609 (16)	0.0473 (14)	0.0280 (13)	−0.0056 (11)	−0.0017 (12)
C7	0.0511 (15)	0.0625 (16)	0.0432 (12)	0.0304 (13)	−0.0069 (12)	0.0017 (13)
C8	0.0491 (15)	0.0436 (14)	0.0383 (12)	0.0255 (12)	−0.0016 (10)	−0.0019 (10)
C9	0.0537 (15)	0.0440 (13)	0.0353 (11)	0.0309 (12)	−0.0025 (10)	0.0000 (10)
C10	0.0600 (17)	0.0442 (15)	0.0621 (16)	0.0338 (14)	−0.0055 (13)	−0.0075 (12)
C11	0.0540 (16)	0.0562 (17)	0.0695 (18)	0.0321 (14)	−0.0018 (13)	−0.0022 (14)
C12	0.0623 (17)	0.0643 (18)	0.0480 (14)	0.0452 (15)	−0.0021 (12)	−0.0023 (13)
C13	0.0733 (19)	0.0496 (16)	0.0658 (17)	0.0430 (16)	−0.0017 (14)	−0.0071 (13)
C14	0.0529 (16)	0.0444 (15)	0.0627 (17)	0.0282 (13)	−0.0049 (12)	−0.0057 (12)
O1	0.0637 (12)	0.0735 (13)	0.0631 (11)	0.0502 (11)	0.0132 (9)	0.0121 (10)
O2	0.0933 (15)	0.0647 (12)	0.0418 (9)	0.0533 (12)	−0.0108 (10)	−0.0124 (9)
S1	0.0604 (4)	0.0513 (4)	0.0401 (3)	0.0394 (3)	0.0020 (3)	−0.0015 (3)
Cl1	0.0746 (5)	0.0945 (6)	0.0928 (6)	0.0635 (5)	−0.0005 (5)	−0.0118 (5)
N1	0.0498 (13)	0.0489 (12)	0.0446 (11)	0.0320 (11)	−0.0005 (10)	−0.0030 (10)
N2	0.0511 (12)	0.0469 (12)	0.0400 (11)	0.0327 (10)	−0.0022 (9)	−0.0032 (9)

### Geometric parameters (Å, º)

**Table:**

C1—C2	1.491 (4)	C8—H8	0.9300
C1—H1A	0.9600	C9—C10	1.374 (3)
C1—H1B	0.9600	C9—C14	1.383 (3)
C1—H1C	0.9600	C10—C11	1.384 (4)
C2—C7	1.381 (3)	C10—H10	0.9300
C2—C3	1.382 (4)	C11—C12	1.369 (4)
C3—C4	1.376 (4)	C11—H11	0.9300
C3—H3	0.9300	C12—C13	1.360 (4)
C4—C5	1.374 (3)	C12—Cl1	1.726 (3)
C4—H4	0.9300	C13—C14	1.373 (4)
C5—C6	1.378 (3)	C13—H13	0.9300
C5—S1	1.751 (3)	C14—H14	0.9300
C6—C7	1.372 (4)	O1—S1	1.4197 (18)
C6—H6	0.9300	O2—S1	1.4189 (19)
C7—H7	0.9300	S1—N1	1.637 (2)
C8—N2	1.265 (3)	N1—N2	1.399 (3)
C8—C9	1.447 (3)	N1—H1	0.875 (17)
			
C2—C1—H1A	109.5	C10—C9—C8	122.5 (2)
C2—C1—H1B	109.5	C14—C9—C8	119.1 (2)
H1A—C1—H1B	109.5	C9—C10—C11	120.9 (2)
C2—C1—H1C	109.5	C9—C10—H10	119.6
H1A—C1—H1C	109.5	C11—C10—H10	119.6
H1B—C1—H1C	109.5	C12—C11—C10	118.8 (3)
C7—C2—C3	118.4 (2)	C12—C11—H11	120.6
C7—C2—C1	121.2 (3)	C10—C11—H11	120.6
C3—C2—C1	120.3 (2)	C13—C12—C11	121.6 (2)
C4—C3—C2	120.9 (2)	C13—C12—Cl1	119.5 (2)
C4—C3—H3	119.5	C11—C12—Cl1	118.8 (2)
C2—C3—H3	119.5	C12—C13—C14	118.8 (2)
C5—C4—C3	119.6 (2)	C12—C13—H13	120.6
C5—C4—H4	120.2	C14—C13—H13	120.6
C3—C4—H4	120.2	C13—C14—C9	121.5 (3)
C4—C5—C6	120.4 (2)	C13—C14—H14	119.3
C4—C5—S1	119.71 (18)	C9—C14—H14	119.3
C6—C5—S1	119.89 (19)	O2—S1—O1	119.65 (12)
C7—C6—C5	119.4 (2)	O2—S1—N1	106.40 (11)
C7—C6—H6	120.3	O1—S1—N1	104.78 (11)
C5—C6—H6	120.3	O2—S1—C5	107.80 (12)
C6—C7—C2	121.2 (2)	O1—S1—C5	109.72 (11)
C6—C7—H7	119.4	N1—S1—C5	107.91 (11)
C2—C7—H7	119.4	N2—N1—S1	113.13 (16)
N2—C8—C9	121.5 (2)	N2—N1—H1	113.4 (17)
N2—C8—H8	119.3	S1—N1—H1	116.2 (17)
C9—C8—H8	119.3	C8—N2—N1	114.7 (2)
C10—C9—C14	118.3 (2)		
			
C7—C2—C3—C4	−0.2 (4)	C11—C12—C13—C14	−1.2 (4)
C1—C2—C3—C4	179.7 (3)	Cl1—C12—C13—C14	176.3 (2)
C2—C3—C4—C5	−0.4 (4)	C12—C13—C14—C9	−0.4 (4)
C3—C4—C5—C6	0.6 (4)	C10—C9—C14—C13	1.8 (4)
C3—C4—C5—S1	−179.42 (19)	C8—C9—C14—C13	−175.3 (2)
C4—C5—C6—C7	−0.1 (4)	C4—C5—S1—O2	−35.4 (2)
S1—C5—C6—C7	179.9 (2)	C6—C5—S1—O2	144.6 (2)
C5—C6—C7—C2	−0.5 (4)	C4—C5—S1—O1	−167.3 (2)
C3—C2—C7—C6	0.7 (4)	C6—C5—S1—O1	12.8 (2)
C1—C2—C7—C6	−179.2 (3)	C4—C5—S1—N1	79.1 (2)
N2—C8—C9—C10	3.7 (4)	C6—C5—S1—N1	−100.9 (2)
N2—C8—C9—C14	−179.3 (2)	O2—S1—N1—N2	57.70 (19)
C14—C9—C10—C11	−1.6 (4)	O1—S1—N1—N2	−174.64 (16)
C8—C9—C10—C11	175.4 (2)	C5—S1—N1—N2	−57.77 (18)
C9—C10—C11—C12	0.1 (4)	C9—C8—N2—N1	−178.3 (2)
C10—C11—C12—C13	1.3 (4)	S1—N1—N2—C8	172.28 (18)
C10—C11—C12—Cl1	−176.2 (2)		

### Hydrogen-bond geometry (Å, º)

Cg is the centroid of the C2–C7 ring.

**Table:**

*D*—H···*A*	*D*—H	H···*A*	*D*···*A*	*D*—H···*A*
N1—H1···O2^i^	0.88 (2)	2.13 (2)	2.952 (3)	156 (2)
C1—H1*A*···O1^ii^	0.96	2.55	3.496 (5)	169
C13—H13···*Cg*^iii^	0.93	2.94	3.823 (3)	160

Symmetry codes: (i) *x*−*y*, *x*, *z*+1/6; (ii) *x*−*y*−1, *x*−1, *z*+1/6; (iii) *x*, *y*+1, *z*.
