# Supplementary figures and images for: Crystal structure of (E)-N′-(4-chloro­benzyl­idene)-4-methyl­benzene­sulfono­hydrazide: a hexa­gonal polymorph
Source: Acta Crystallogr Sect E Struct Rep Online. 2014 Nov 12;70(Pt 12):o1250–1. doi: 10.1107/S1600536814023721 (PMC4257446; doi:10.1107/S1600536814023721)

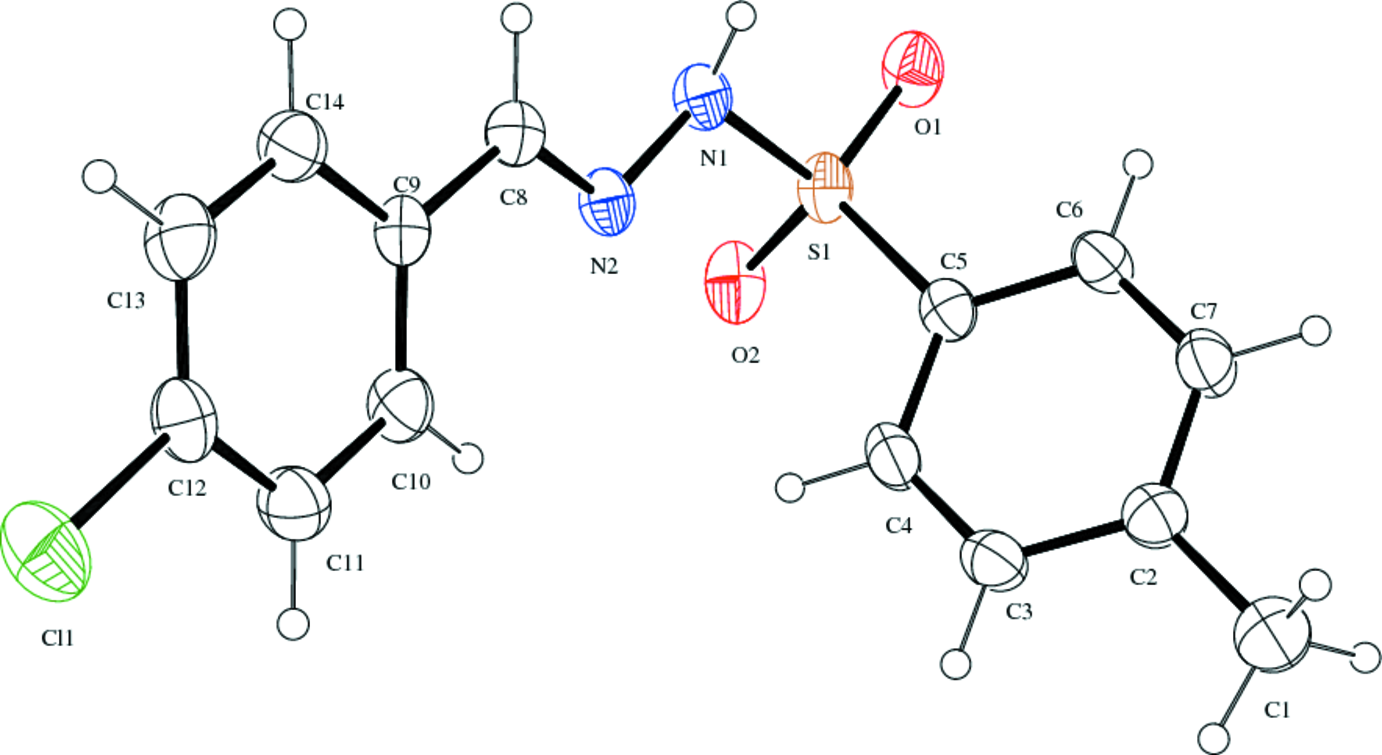

Supplement: Supplementary file 4 [file e-70-o1250-fig1.tif]

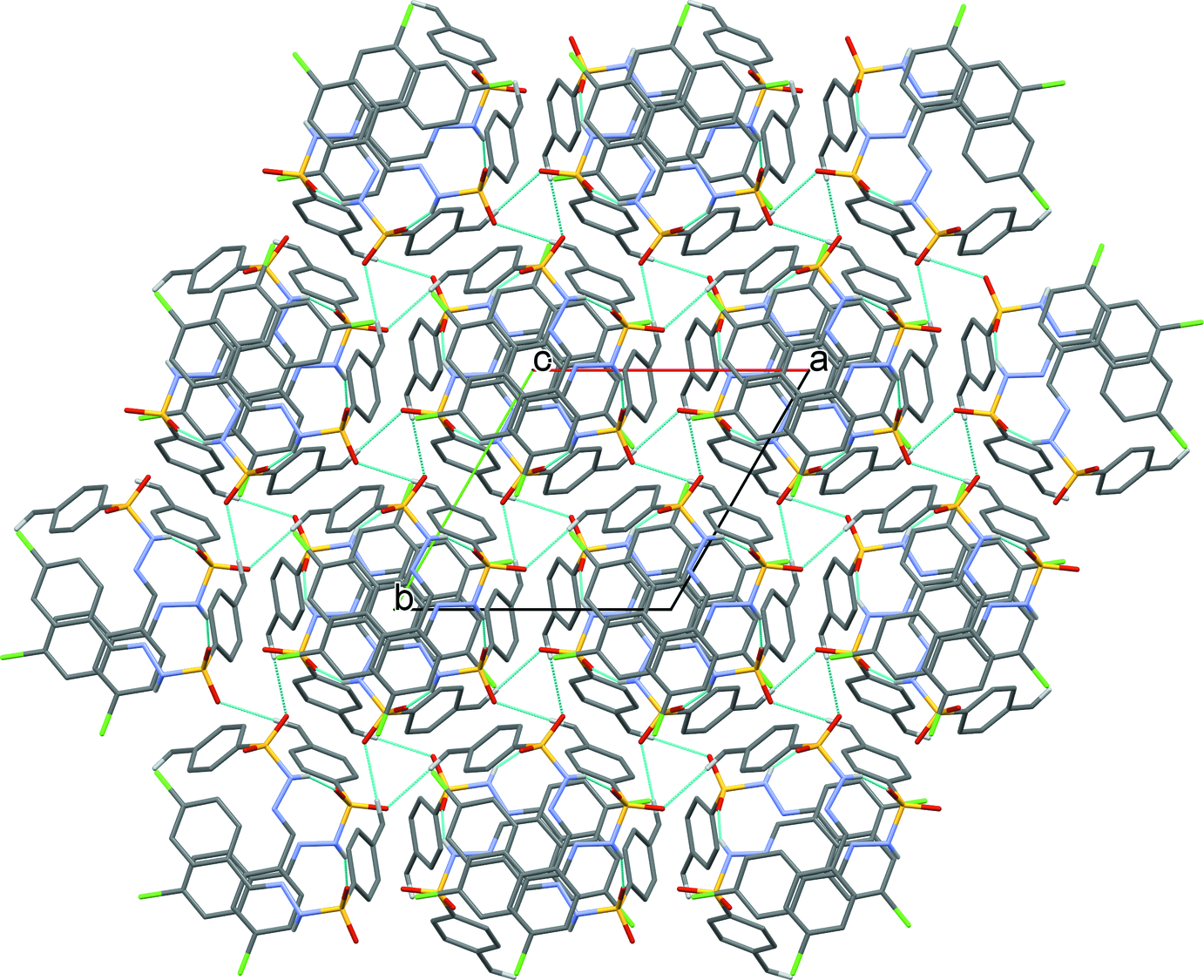

Supplement: Supplementary file 5 [file e-70-o1250-fig2.tif]
